# Stability and Maintenance of Foxp3^+^ Treg Cells in Non-lymphoid Microenvironments

**DOI:** 10.3389/fimmu.2019.02634

**Published:** 2019-11-14

**Authors:** Thomas Korn, Andreas Muschaweckh

**Affiliations:** ^1^Department of Experimental Neuroimmunology, Klinikum rechts der Isar, Technical University of Munich, Munich, Germany; ^2^Department of Neurology, Klinikum rechts der Isar, Technical University of Munich, Munich, Germany; ^3^Munich Cluster for Systems Neurology (SyNergy), Munich, Germany

**Keywords:** Treg—regulatory T cell, Foxp3, heterogeneity, central nervous system, stability, non-lymphoid tissues

## Abstract

Foxp3^+^ Treg cells are indispensable for maintaining self-tolerance in secondary lymphoid organs (SLOs). However, Treg cells are also recruited to non-lymphoid tissues (NLTs) during inflammation. Recent advances in the understanding of Treg cell biology provided us with molecular mechanisms—both transcriptional and epigenetic—that enable Treg cells to retain their identity in an inflammatory milieu that is *per se* hostile to sustained expression of high levels of Foxp3. While Treg cells are recruited to sites of inflammation in order to resolve inflammation and re-establish appropriate organ function, it is increasingly recognized that a series of inflammatory (but also non-inflammatory) perturbations of organ function lead to the constitution of relatively long lived populations of Treg cells in NLTs. NLT Treg cells are heterogeneous according to their respective site of residence and it will be an important goal of future investigations to determine how these NLT Treg cells are maintained, e.g., what the role of antigen recognition by NLT Treg cells is and which growth factors are responsible for their self-renewal in the relative deficiency of IL-2. Finally, it is an open question what functions NLT Treg cells have besides their role in maintaining immunologic tolerance. In this review, we will highlight and summarize major ideas on the biology of NLT Treg cells (in the central nervous system but also at other peripheral sites) during inflammation and in steady state.

## Introduction

Foxp3^+^ Treg cells have been intensely studied over the last three decades (1). Treg cells in secondary lymphoid tissues are indispensable for maintaining immune tolerance because elimination of Treg cells either in newborn individuals or in adults results in multiorgan autoimmunity within a couple of weeks (2, 3). It is an intriguing concept to exploit Treg cells for therapeutic interventions—either enhancing their function in autoimmunity or dampening their effect in cancer. Proof of concept trials in graft-vs.-host disease and even in type 1 diabetes have been undertaken in humans (4, 5). However, antigen specificity of Treg cells (most trials were performed with polyclonal Treg cell populations), their trafficking behavior, and their stability after adoptive transfer into human hosts remain challenges on the way to a broader application of adoptive Treg cell therapy (6). Perhaps it is now time to take a step back and consider novel and unconventional concepts about Foxp3^+^ Treg cells that might nevertheless be fundamental to the understanding of Treg cells in homeostasis and in disease conditions: First, Treg cells might have some plasticity and adapt to the functional context, in which they need to be operational (7). For instance, during inflammation, Foxp3^+^ Treg cells upregulate pathways that secure the preservation of their identity as Treg cells but might have additional (as yet unknown) functions for the establishment of tissue-resident Treg cell subsets. Second, Treg cells do not only reside in secondary lymphoid tissue but also in NLT. These “tissue-resident” Treg cells are distinct from circulating lymphoid tissue Treg cells and might in some cases populate distinct tissue niches (8). Limited information exists as to whether tissue-resident Treg cells are differentially recruited from the systemic repertoire or whether their functions are imprinted *in situ* in their particular niche. Also, their TCR repertoire and the role of antigen for their maintenance is not known. Finally, they might exert “non-canonical” functions in these tissues that do not have anything to do with the regulation of immune responses in the first place but with tissue development and organ homeostasis. In this review, we will discuss some of these aspects in the central nervous system (CNS) and in those peripheral organs where Treg cells have been investigated in non-lymphoid tissue niches.

## Stability of Foxp3 Treg Cells in the CNS in the Context of Autoimmunity

Treg cells are crucial for the regulation of autoimmune inflammation in the CNS. Depletion of Treg cells lowers the threshold for autoimmune CNS inflammation in individuals whose T cell receptor repertoire contains large fractions of CNS reactive T cells (9). Moreover, depletion of Treg cells prior to or after onset of experimental autoimmune encephalomyelitis (EAE) worsens the disease and prevents recovery (10–12).

Since it is clear that Foxp3^+^ Treg cells are recruited to the target tissue of autoimmune reactions not only in the CNS (13, 14) but also in other organs including the joints (15), the pancreas (16), or the skin (17, 18), a major area of investigation in Treg cell biology in the recent years has been their stability in an inflammatory environment. Since it has been recognized that Foxp3^+^ Treg cells are recruited directly to the site of inflammation, Treg cells must dispose of active mechanisms of resilience to maintain their functional phenotype in spite of inflammatory cues in their environment. A variety of pathways have been described, which all ultimately result in keeping the expression of Foxp3 at high levels when factors of the inflammatory milieu activate pathways that otherwise would destabilize Foxp3 expression. The overarching concept is that Foxp3 interacts with (16–19) or is co-expressed with various combinations of transcription factors in Treg cells to induce an effector Treg (eTreg) program and to adapt to the quality of the inflammatory response that is supposed to be controlled by these Treg cells (19–21) while at the same time preserving their identity as Treg cells. Here, direct transactivators of Foxp3 as well as transcriptional inhibitors of effector T cell programs have been described (Table 1).

**Table 1:** Selection of molecules directly involved in the transcriptional regulation of Foxp3 in murine NLT Treg cells.

**Modulator**	**Mechanism**	**References**
Runx-CBFβ	Occupation of *Foxp3* promoter and CNS2. Also relevant for steady-state Foxp3 expression.	(22)
Foxp1	Foxp1 co-occupies Foxp3 target loci. Negative regulation of Satb1 expression in Treg cells.	(23)
HIF1α	Exaggerated expression of HIF1α in Treg cells (by ablation of the E3 ubiquitin ligase VHL) leads to their metabolic reprogramming into effector T cells.	(24)
DBC1	DBC1 physically interacts with Foxp3 and renders the complex more susceptible to inflammation induced degradation.	(25)
Pak2	Treg cells deficient in p21-activated kinase 2 (Pak2) convert into Th2 cells with high Gata3 expression.	(26)

Moreover, the significance of epigenetic modifications both of the chromatin in the vicinity of the Foxp3 locus and of the Foxp3 locus itself in regulating the expression of Foxp3 in distinct milieus is increasingly appreciated (27, 28). In addition to the promoter of Foxp3, three conserved non-coding regions (*CNS1-3*) have been identified in the Foxp3 locus, whose methylation status determines the efficacy with which *Foxp3* is transcribed since for instance, Ets-1 transcription factors only bind to *CNS2* [i.e., the conserved non-coding sequence in the first intron of the *Foxp3* locus that has also been termed Treg specific demethylated region (TSDR) (29)] in its demethylated state and thus increase the enhancer activity of *CNS2* for *Foxp3* (30).

During local inflammation, the central nervous system milieu represents a particular challenge to the identity and function of eTreg cells. The most relevant molecular mechanisms that preserve the “identity” of Treg cells (e.g., their sustained expression of Foxp3) have been a matter of debate. Recently, it has been shown that both TCR/Irf4 signaling and NFκB signaling are required independently of each other to establish the eTreg cell transcriptional program (31, 32); and the transcriptional modifier Blimp1 is a master controller of the eTreg program in Treg cells (33). Loss of Blimp1 in Treg cells in steady state does not produce an inflammatory phenotype, most likely due to the fact that steady state Treg cells in secondary lymphoid tissue only express low levels of Blimp1. In contrast, loss of Blimp1 in Treg cells in the inflamed CNS or in the colon has major consequences: First, these NLT Treg cells lose their effector Treg phenotype. For instance, since Blimp1 is a direct transactivator of *Il10*, Blimp1 deficient Treg cells are unable to produce IL-10 (34–36). Second, Blimp1 is also required to maintain the very identity of Treg cells in an inflammatory milieu and loss of Blimp1 eventually leads to downregulation of Foxp3 expression (35). Mechanistically, *Foxp3* is not a transcriptional target of Blimp1. Rather Blimp1 appears to be controlling the expression of Foxp3 in an indirect manner by preventing the inflammatory environment (and particularly IL-6) from methylating *CNS2*. In fact, Blimp1 suppresses the expression of the methyl transferase Dnmt3a in Treg cells in an inflammatory environment (35).

In principle, genomic CpG islands are methylated and demethylated by DNA methyltransferases and Tet methylcytosine dioxygenases, respectively (37). The modulation of the demethylating enzymes Tet2 and Tet3 in Treg cells affects *CNS2* methylation and Treg cell stability (38). Notably, the activity of the Tet enzymes might be controlled by intermediates of the mitochondrial tricarboxylic acid cycle. In fact, ablation of the mitochondrial transcription factor A (Tfam) leads to altered succinate/α-ketoglutarate and fumarate/α-ketoglutarate levels in Treg cells—and as a consequence—to reduced activity of the Tet enzymes and a failure to maintain the demethylated state of *CNS2* (39). By this mechanism, intermediary metabolism in Treg cells is coupled to their stability. Conversely, DNA methyltransferases (and in particular Dnmt1 and Dnmt3a) are able to methylate *CNS2* (and other CpG islands in the *Foxp3* locus) and thus dampen Foxp3 expression. Here, an interesting model has recently been suggested (40): STAT5 (downstream of IL-2) binds to *CNS2* irrespectively of the methylation status of *CNS2* and increases its enhancer activity as to the expression of Foxp3 (41). Both STAT3 (downstream of IL-6) and STAT6 (downstream of IL-4) can compete with STAT5 for its binding sites in *CNS2*. In contrast to STAT5, both STAT3 and (more prominently) STAT6 appear to physically interact with Dnmt1 and Dnmt3a (40, 42), and thus can mediate their recruitment to *CNS2*. Methylation of *CNS2* by these methyl transferases would then lead to the silencing of *Foxp3*. This model provides a rationale for inflammation (IL-4 and IL-6) induced downregulation of Foxp3 in Treg cells and IL-2 dependent counteraction of this process.

Apart from epigenetic modulation of the *Foxp3* locus itself, histone modifications also control Foxp3 transcription. Interestingly, partial methylation of *CNS2*, which occurs in Treg cells exposed to an inflammatory environment, results in the recruitment of methyl CpG binding protein 2 (MeCP2) to the methylated CpG islands of *CNS2*. MeCP2 in turn leads to the acetylation of H3, which reinforces Foxp3 transcription (43). Also, Ezh2, a chromatin modifying enzyme that catalyzes the trimethylation of H3K27, is co-expressed in Treg cells in response to CD28 stimulation. Foxp3 cooperates with Ezh2 to repress its target genes, and in the absence of Ezh2, Foxp3 is still present in Treg cells but fails to reinforce its transcriptional program (44).

Taken together, the stability of Foxp3^+^ Tregs at sites of inflammation is instructed by an active process. Treg cell extrinsic cues (TCR stimulation, co-stimulation, cytokines) are required to secure the stability of Treg cells and both direct transcriptional as well as epigenetic mechanisms are involved in maintaining high levels of Foxp3 expression and thus the functional phenotype of Treg cells in inflamed tissues.

## Maintenance of NLT Foxp3^+^ Treg Cells During and After Inflammation

Compelling evidence suggests that IL-2 is a non-redundant growth factor of Treg cells in secondary lymphoid tissues. Treg cells do not produce IL-2 themselves but rely on extrinsic sources of IL-2, which is mostly provided by conventional T cells. Lack of IL-2 leads to the attrition of Treg cells and to the development of multi-organ autoimmunity (45). Mechanistically, IL-2 counteracts the apoptosis prone transcriptional program of Treg cells (46). It is quite likely that during organ specific inflammation—due to massive infiltration of conventional T cells—the source of IL-2 is sufficient also in NLTs to fuel the maintenance and even expansion of Foxp3^+^ Treg cells (47). In fact, a decrease in the threshold of IL-2 responsiveness in Treg cells or more efficient STAT5 signaling, which can be modulated by a plethora of mechanisms (23, 40, 48), have been identified as a key “stability” mechanism of Treg cells in NLTs under conditions of limited availability of IL-2 (see above).

**Figure 1:**
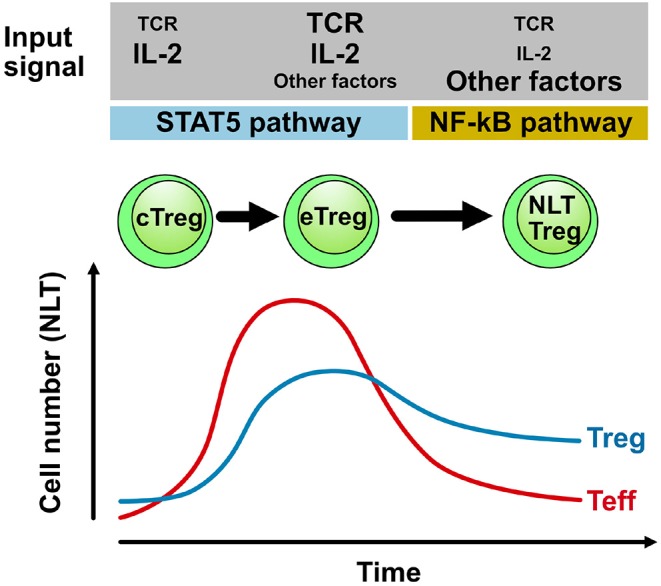
Population dynamics and maintenance of Treg cells in NLTs. During an adaptive immune response in NLT (e.g., in the central nervous system during EAE) conventional T cells (Teff) infiltrate the target tissue and expand providing sufficient IL-2 to also drive the differentiation and expansion of Treg cells. After contraction of the conventional effector T cell (Teff) population (due to active regulation by Treg cells), IL-2 becomes limiting and persisting Treg cells likely depend on alternative signals for self-renewal. Here, signals that activate the NF-κB pathway might be important cues not only for NLT function but also for their maintenance (31). TCR triggering of Treg cells and downstream modulation of the TCR signal by the transcriptional modulator IRF4 is a non-redundant event in the differentiation of central Treg cells (cTreg) in secondary lymphoid tissues into effector Treg cells (eTreg) in the inflamed tissue. The requirement of the TCR signal and other cues in NLT Treg cells that reside in specific niches over extended periods of time needs to be determined and likely depends on the anatomical niche that might then also drive a distinct functional specialization of NLT Treg cells.

However, after the contraction of the effector T cell population in the target tissue of the inflammation, the source of IL-2 (and thus the relevant growth factor for Treg cells) becomes limiting. As a consequence, the Treg cell population in NLTs will also contract (47). However, we and others have observed that after a traumatic or inflammatory event in the central nervous system (but also in other tissues), Foxp3^+^ Treg cells remain in the central nervous system for extended periods of time and might even establish a population of resident Treg cells in the relative absence of conventional T cells (13). Little is known about this “meta-homeostatic” Treg cell population (Figure 1). Previous studies suggested that Treg cells which infiltrate the CNS during EAE were most likely exclusively thymus-derived and not peripherally induced from conventional T cells (13). In particular, as mice, which are unable to generate peripherally induced Treg cells due to a mutation of the *Foxp3* locus lacking *CNS1* but have regular numbers of thymus derived Treg cells, do not have an EAE phenotype, Treg cells in the inflamed central nervous system must be thymus-derived (49). However, it is an open question how this NLT Treg cell population is selected or whether its specific properties are locally imprinted and also how it is maintained.

**Figure 2:**
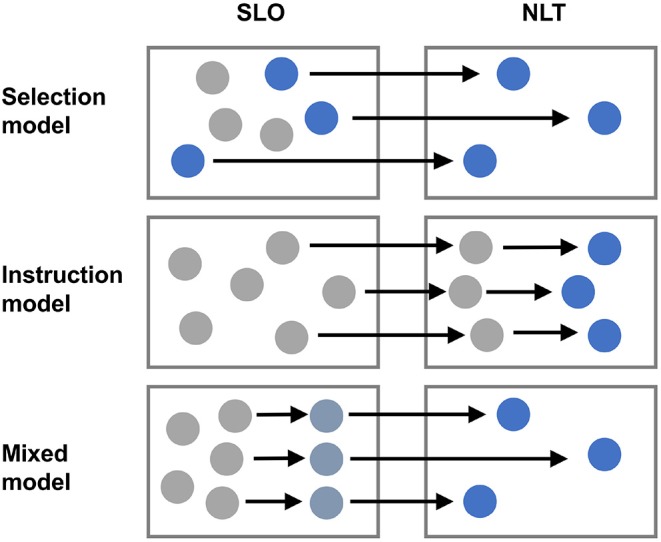
Heterogeneity of Treg cells in NLTs. Treg cells are heterogeneous and the NLT milieu is likely an important determinant of this heterogeneity. However, it is unclear whether specific hard-wired subsets of Treg cells are selectively recruited from SLOs to distinct NLTs (selection model) or whether Treg cells that enter a given NLT are locally instructed to adopt specific properties and specialize according to a niche specific program (instruction model). Alternatively, some degree of imprinting might occur in SLOs during priming and further commitment of Treg cells to certain tissue specific properties might then take place in NLTs (mixed model).

In skin and colon NLT Treg cells have been characterized by scRNAseq as to a specific “barrier tissue” transcriptional program and as to their provenance and development (in pseudotime analyses) (50). By analyzing NLT Treg cells, it has been possible to define a transcriptional program reminiscent of the effector Treg (eTreg) phenotype that has been coined for Treg cells isolated from the visceral adipose tissue (33, 51). While definitive proof by provenance mapping systems is still lacking, based on these scRNAseq analysis, the instruction of a Treg cell trait consistent with tissue residency might be initiated in secondary lymphoid tissues. Further imprinting of functional states then occurs in the NLT (Figure 2). Best evidence for this model has been presented for VAT Treg cells: By using PPAR-γ reporter mice, a previous study has identified a small population of PPAR-γ^low^ expressing Treg cells in secondary lymphoid organs (SLO), in which part of the VAT Treg transcriptional program, in particular those genes associated with Treg activation, was already active, and these cells were shown to be capable of adopting the full VAT Treg signature upon their migration into the tissue (52), most likely in response to local tissue-specific cues. Based on this finding, it was proposed that the bona-fida VAT Treg gene signature/phenotype might be adopted in a sequential, step-wise process, that is initiated in the SLO and then finalized within the non-lymphoid compartment. The first step appears to be a “priming” step in SLOs, where Treg cells receive an activating cue, e.g., in response to cognate self antigen recognition. Then they gain the capacity to infiltrate into NLTs. In a second step, Treg cells that re-encounter their specific antigen in the NLT, might be preferentially retained *in situ* and receive further tissue-specific cues that impart the full NLT phenotype and function (52).

In particular, NFκB (RelA) signaling appears to be a hallmark of the eTreg cell [and NLT Treg cell) program. Whether TNF family members (including TNF-ligand related molecule 1 (31, 53)] or IL-1 family members [including IL-33 (51) and IL-18 (54)] are more crucial or differentially important to guide the NLT Treg program, remains to be determined. Also, it remains to be determined whether NFκB signaling into Treg cells secures their maintenance outside SLOs where IL-2 derived from conventional T cells, which is a major nutrient of Treg cells in lymphoid tissues, is scarce.

Very little is known about the “Treg cell growth and maintenance” factors in NLTs and whether they are universal for all peripheral tissues or private to distinct anatomical niches. The alarmin IL-33, a member of the IL-1 family of cytokines, plays a major role in driving the expansion/accumulation of VAT-resident Treg cells (51, 52, 55). IL-33 is constitutively expressed in the CNS even under physiological conditions (56). Given the high expression of its receptor ST2 (encoded by *Il1rl1*) on brain Treg cells and the observation that Treg cells fail to properly expand in the injured ischaemic brain in ST2- and IL-33-deficient mice (57), IL-33 might be a prominent candidate capable of substituting for the role IL-2 in mediating Treg cell survival in the post-inflammatory central nervous system. Another such candidate could be the neurotransmitter serotonin (5-HT). Its receptor 5-HT7 protein (encoded by *Htr7*) is specifically upregulated on Treg cells accumulating in the ischaemic brain (57), and it was demonstrated that both serotonin or the inhibition of its uptake could expand brain Treg cell numbers *in vivo*.

In summary, NLT Treg cells (in the central nervous system but also in other tissues) likely populate niches that provide a distinct environment for their survival. The molecular pathways that support their survival (and possible self renewal) are likely different from the IL-2 driven Treg cell maintenance system in secondary lymphoid organs.

## Non-Canonical Functions of Treg Cells

While thymus-derived Treg cells that populate SLOs are non-redundant in maintaining self tolerance, eTreg cells in NLTs also clearly regulate effector responses of conventional T cells during adaptive immune responses in order to clear inflammation and reduce immune pathology. However, since Treg cells are found in NLTs in steady state and after re-establishment of homeostasis after any kind of injury (meta-homeostasis), it has been suggested that tissue intrinsic (non-immune) functions of these peripheral tissues might be controlled by local Treg cells (8).

Various NLTs, including barrier tissues like the skin, the colonic lamina propria and the lung, as well as non-barrier sites, like the visceral adipose tissue (VAT) of lean mice or the skeletal muscle are populated by a distinct population of regulatory T cells. As outlined above, NLT Treg cells show a high degree of phenotypic and functional adaptation to the NLT environment in which they reside and share a set of common NLT-specific transcripts that distinguish them from their counterparts in SLOs. The vast majority of NLT Treg cells display an elevated expression of genes typically associated with an eTreg phenotype, such as the effector molecules Ctla4, Icos, Klrg1, and GzmB. Despite a certain overlap in the transcriptomes between NLT Treg cells from different NLTs, there are a number of transcripts specifically and distinctively upregulated in Treg cells from particular non-lymphoid compartments, which might endow these NLT Treg cells with specialized functions in organ homeostasis.

For example, VAT Treg cells highly express the transcription factor peroxisome proliferator-activated receptor (PPAR)-γ, the master regulator of adipocyte differentiation. PPAR-γ governs much of the unique transcriptional signature of VAT Treg cells (58), including the expression of genes involved in lipid metabolism, such as *Dgat1* (diacylglycerol O-acyltransferase 1), encoding an enzyme involved in triacylglycerol biosynthesis, *Pcyt1a* (choline-phosphate cytidylyl transferase A), which encodes an enzyme involved in phosphatidylcholine synthesis, as well as *Cd36*, encoding the lipid scavenger receptor CD36 (59). Specific deletion of PPAR-γ in Treg cells (using Foxp3-Cre x *Pparg*^flox/flox^ mice) impairs their accumulation in VAT, but not in SLOs, and promotes an increased inflammatory state in the VAT associated with enhanced systemic insulin resistance (59).

In other NLTs, further functions of resident Treg cells have been described that are unrelated to the control of immune responses in the first place. For example, in the steady state skin, Foxp3^+^ Treg cells are localized in close proximity to hair follicles where they promote the proliferation of hair follicle stem cells and the telogen to anagen transition of the hair follicle by providing the Notch-ligand Jag1, which is sensed by hair follicle stem cells (60). In lung tissue and in muscle tissue, Treg cells contribute to tissue regeneration after injury by producing the epidermal growth factor receptor ligand amphiregulin, to which bronchial epithelial cells respond with proliferation and differentiation (54) and muscle satellite cells respond with myogenic differentiation (61), respectively.

In contrast to NLT such as the VAT, intestine, lung or skin, “immune-privileged” compartments like the central nervous system are largely devoid of Treg cells during steady state conditions. However, Treg cells readily accumulate in the CNS in response to acute local injury (e.g., hypoxia/stroke) (62) or autoimmune inflammation (13). Interestingly, Treg cell numbers remain elevated in the post-EAE central nervous system for a prolonged period of time, raising the question of whether these cells might also play a significant role in promoting tissue repair in the recovering central nervous system. In fact, Treg cells have already been shown to be capable of supporting myelin regeneration by promoting the differentiation of oligodendrocyte progenitor cells and their production of myelin in both the brain and the spinal cord *in vivo* in a model of toxic demyelination (lysolecithin or cuprizone injection) (63). Central nervous system Treg cells have a transcriptional profile that clearly differs from that of SLO Treg cells (35, 57), and in many ways resembles the profile of other NLT Treg populations. Microarray analysis of Treg cells that had infiltrated the brain after acute ischaemic injury revealed that these Treg cells appear to be transcriptionally related to VAT and skeletal muscle Treg cells, as demonstrated by the high level expression of genes that encode IL-10, amphiregulin (*Areg*); Klrg1, ST2, and PPAR-γ. In addition, brain Treg cells upregulate certain central nervous system-specific genes, such as neuropeptide Y (*Npy*), preproenkephalin (*Penk*), serotonin receptor type 7 (*Htr7*), and arginine vasopressin receptor (*Avpr1a*) (57). In particular the increased expression of the epidermal growth factor ligand amphiregulin by CNS recruited Treg cells seems important for neurological recovery as demonstrated by its capacity to suppress astrogliosis, neurological deficits and neurotoxic gene expression (57).

Therefore, NLT Treg cells might be private to their own niche of residence and intricately connected to tissue intrinsic non-immune processes in homeostasis and organ development.

## Human NLT Treg Cells

NLT Treg cells have been extensively studied in mice, however it has become clear that essentially all human NLTs also harbor a distinct population of Treg cells (64, 65). The functional phenotype of human NLT Treg cells remains largely unexplored and it is possible that pathways identified as relevant for the maintenance and function of murine NLT Treg cells may not have the same importance for human NLT Treg cells. For example, omental fat tissue—the human correlate of murine VAT—has been reported to contain detectable levels of Foxp3 transcripts (58). Like their counterparts in mice, omental fat Treg cells have been shown to express ST2 (51) while other studies failed to detect ST2 expression on Foxp3^+^ Treg cells isolated from human omental adipose tissue and also other organs including colon and lung (66, 67), suggesting that the IL-33/ST2 axis may not be a universal pathway for the maintenance of human NLT Treg cells.

Treg cells are also abundant in healthy adult human skin, comprising about 20% of the total CD4^+^ T cell population. Human skin Treg cells exhibit an activated memory phenotype, as defined by their almost uniform expression of the memory markers CD45RO, CD27, and Bcl-2 and the increased expression of Foxp3 and Treg activation markers, such as CTLA-4, CD25, and ICOS relative to their counterparts in the peripheral blood. There is little overlap in the TCR repertoire between Treg cells and conventional T cells in healthy human skin, suggesting that these two NLT T cell populations recognize different antigens, similar to what has been observed for Treg cells and conventional T cells from murine VAT (68). Interestingly, similar to their murine equivalents (60), dermal Treg cells in normal human skin were found to preferentially reside in close proximity to hair follicles (68).

The currently available data suggest a considerable overlap in the expression of tissue-specific Treg cell markers between mice and humans, as demonstrated for skin- or gut-derived Treg cells (50). Also, the NLT core signature which is characterized by an enrichment of genes related to the TNFRSF-NF-κB pathway appears to be largely conserved between mouse and human Treg cells (50, 69), raising the possibility that human NLT Treg cells might undergo a similar process of local imprinting and tissue adaptation during their establishment in NLTs.

## Perspective

Fundamental questions regarding NLT Treg cells are as yet unresolved: For example, during organ specific inflammatory responses, Treg cells that are recruited to the sites of inflammation are faced with issues of stability of their phenotype and deficiency in growth factors that they would otherwise be able to rely on in steady state in SLOs. Also, it is unclear whether subsets of Treg cells are pre-committed to certain phenotypes in the secondary lymphoid system and are then selectively recruited to certain anatomical niches where they expand or whether “naive” Treg cells are locally instructed to adopt a distinct phenotype only after the recruitment to specific organ systems. Answers to these questions will profoundly affect concepts of therapeutic interventions that intend to make use of tissue-resident Treg cells. It might in fact be oversimplified (and perhaps even dangerous) to aim for a “one-size-fits-all” Treg cell. Rather, more tailored strategies to either selectively expand subsets of Treg cells or to instruct them to adopt appropriate (organ-specific) features might have to be developed. Here, we face dramatic gaps in knowledge and a renewed interest in Treg cell centered therapeutic approaches that exploit the immune functions of Treg cells needs to consider the emerging profound organ specific heterogeneity of Foxp3^+^ Treg cells and their potential functions in tissue homeostasis and regeneration.

## Author Contributions

TK conceptualized and wrote the review. AM wrote parts of the review and edited the text.

### Conflict of Interest

The authors declare that the research was conducted in the absence of any commercial or financial relationships that could be construed as a potential conflict of interest.
